# Sloughy Abscess of the Leg, &c.

**Published:** 1830-07-01

**Authors:** 


### ST. GEORGE'S HOSPITAL.

SLOUGHY ABSCESS OF THE LEG, SUPERVENING ON OTHER DISEASES, AND OCCASIONALLY ATTENDED WITH INFLAMMATION OF THE LARGE NERVES.

IT appears that unhealthy suppuration in the leg not unfrequently arises in the progress of fever or febrile affections, after injuries of the head, fractures in the extremities, and, in short, from a variety of causes. We are not aware that writers have particularly noticed these cases, but we have reason to believe that they are very frequent, the symptoms are peculiar and severe, and the result not seldom fatal. It is proper that practitioners should be acquainted with the nature and existence of this affection, for there is only one successful mode of treatment, and unless that be speedily adopted disastrous consequences should be seriously apprehended. Without any further preliminary remarks, we shall mention the particulars of some of the cases which have fallen under our observation.

CASE 1. *Menorrhagia—Swelling of the Leg—Sloughy State of the Posterior Tibial Nerve.*—On the 6th of last June, one of the dressers of the hospital was requested to see a woman of the name of Sarah Lazenby, about sixty years of age. He found her in a filthy hovel in Westminster, labouring under typhoid symptoms, with slight mænorrhagia, and pain in the head and back. It was about "the turn of life," and until a fortnight previously she had missed the catamenia for three months. At that time, however, she had been attacked with mænorrhagia, which lasted for four or five days, then ceased, and again returned two or three days before the dresser's visit, when it was nearly suppressed, as has been mentioned.

Next day, 7th, she complained of great pain and tenderness on pressure in the right leg, which was swollen at the calf. On the following evening, 8th, she was received in the hospital under Dr. Seymour. The treatment had consisted of salines with the liquor opii sedativus.

On her admission she was evidently in a state of great exhaustion. The leg was much swollen at the calf and exquisitely painful, but although very tense, no distinct fluctuation could be felt. On the 9th she died.

*Sectio Cadaveris.* Body not much emaciated. Right leg still swollen, especially about the upper part of the calf. On making an incision on its inner side, a considerable quantity of bloody serum was found in the subcutaneous and inter-muscular cellular membrane—the gastrocnemius and soleus were swollen and somewhat tense, with small coagula of recent blood effused between their fasciculi—and the cellular membrane between the soleus and the deep-seated layer of muscles was infiltrated with serum, some blood, and in two or three places an attempt at pus. The posterior tibial nerve in the upper third of the leg was enlarged, dark-looking, injected, inflamed, and evidently implicated in the commencing sloughing of the cellular membrane. The veins were perfectly healthy.

*Abdomen.* Uterus as large as in the second month of pregnancy—its cavity of considerable size, with coagula of blood attached to its mucous membrane, which was rough at the fundus, vascular, and shaggy. Parietes of the uterus rather flabby—vagina healthy.

*Cranium.* Much venous turgescence, and good quantity of serous effusion between the membranes and in the ventricles.

CASE 2. *Fever—Sloughy Abscess of the Leg—Implication of the Posterior Tibial Nerve.* A very similar case to the preceding was lately under the care of Dr. Seymour and Mr. Hawkins in the hospital. The patient was labouring under fever, and in a low state, when rather suddenly the leg swelled, became extremely painful, and appeared to be rapidly running into mortification or sloughing. Deep-seated fluctuation being felt an incision was made through the fascia, when a quantity of stinking pus was discharged. The patient experienced temporary relief, but sank in a few days.

On examination there was found a large, deep, sloughy abscess in the calf, and the posterior tibial nerve was involved in the inflammation. It was swollen and enlarged for an inch or two in length, injected, dark, and sloughy looking.

In both the foregoing cases the patients had suffered exquisite pain, and the nervous system was excessively depressed, with genuine typhoid symptoms. We remember having witnessed two or three years ago a case resembling the former in some respects. The patient was a young man under the care of the late Mr. Rose, and we think had undergone some trifling operation. Suddenly an abscess formed in the leg, accompanied with low constitutional disturbance, and although the matter was discharged by free incisions, purulent depôts took place in the lungs, and of course proved fatal.

Case 3. *Blow upon the Head—Epistaxis—Abscess in the Legs.*—John Sterden, æt. 27, a fishmonger, admitted April 28th, 1830, under Mr. Keate.

About four months ago he fell against a wall, and struck his head, but not severely. Two or three days afterwards the nose bled to a very considerable amount, which it did for a second and a third time. He was much debilitated and confined to his room in consequence, and in a fortnight after the epistaxis a swelling made its appearance in the calf of the left leg. Has never had rheumatic symptoms.

At present there is evidently an abscess of some extent, rather deeply seated in that situation—manner very quick and anxious—face pallid—health indifferent.

On the 1st of May an opening was made with a lancet at the upper part of the swelling, and much matter was discharged. In about a month another incision was made below and more matter issued. Pain now attacked the other leg and leeches were applied; the leech-bites festered; a poultice was necessary, and afterwards a roller which was drawn too tight by the dresser and produced considerable swelling of the leg. Matter formed in various parts and required incisions; one four inches long on the inner and upper part of the leg, another about its middle on the outside, and a third in the calf; pus was freely discharged for some time from all. Aperient medicines, &c. were exhibited, and he was put upon sarsaparilla and nitric acid. The suppuration in the left leg soon ceased, and the limb was strapped and bandaged. On the 4th of June the right leg was bandaged also, and at present, 20th, there is very little secretion of pus, although a good deal of tumefaction remains, and some pain. His health is improved.

Case 4. *Fracture of the Leg—Abscesses in the same—Sloughy Abscess in the opposite Leg.*—Thomas Risley, æt. 45, a publican, admitted June 9, 1830, under Mr. Hawkins.

In the calf of the left leg a large abscess, evidently deeply seated—much pain—skin not affected—aspect sallow—health indifferent.

Nine months ago broke his right leg near the ankle, and whilst lying in bed for the accident two abscesses formed in succession in the same leg. When he was recovering from these he was attacked with "ague," and after he had recovered from this with "inflammation of the liver." No sooner had this been dispersed than the present abscess in the left leg appeared, which is now about four months ago.

On the 10th the abscess was opened by cautious and deep incisions with a scalpel and director through the fascia. A considerable quantity of thin, dirty, bloody-looking pus was evacuted. Poultices, salines, and subsequently decoction of bark was employed, the health is surprisingly improved, and the tumefaction of the leg much diminished.

We might give one or two more cases of the same description, but enough has been said to shew that abscess in the calf of the leg is a frequent affection, that it generally occurs in cachectic or debilitated patients, and that sometimes it puts on so severe a form as to prove the cause of death. We have also shewn that the posterior tibial nerve may become implicated, and that the symptoms are then proportionably severe. With regard to the treatment, there can be no question that early and deep incision, through the fascia if necessary, is the main thing to be depended on. The pus being discharged, the general health is to be attended to, and is greatly benefited by sarsaparilla or tonics, as the case may require.

---

